# Supplementary material for: Cellular geometry and epithelial-mesenchymal plasticity intersect with PIEZO1 in breast cancer cells
Source: Commun Biol. 2024 Apr 17;7:467. doi: 10.1038/s42003-024-06163-z (PMC11024093; doi:10.1038/s42003-024-06163-z)
Supplement: Supplementary file 2 — Supplementary Information [file 42003_2024_6163_MOESM2_ESM.pdf]

Cellular geometry and epithelial-mesenchymal plasticity intersect with PIEZO1 in breast cancer cells

Choon Leng So[1,2], Mélanie Robitaille[1], Francisco Sadras[1], Michael H. McCullough[3,4], Michael J. G. Milevskiy[5,6], Geoffrey J. Goodhill[3,7], Sarah J. Roberts-Thomson[1], Gregory R. Monteith[1*]

[1]School of Pharmacy, The University of Queensland, Woolloongabba, QLD, Australia 4102.
[2]Present address: Department of Biochemistry and Molecular Biology, Johns Hopkins Bloomberg School of Public Health, Johns Hopkins University, Baltimore, MD 21205, USA.
[3]Queensland Brain Institute and School of Mathematics and Physics, The University of Queensland, Brisbane, QLD, Australia 4072.
[4]Present address: Eccles Institute of Neuroscience, John Curtin School of Medical Research, and School of Computing, ANU College of Engineering and Computer Science, The Australian National University, ACT, Australia 2600.
[5]ACRF Cancer Biology and Stem Cells Division, The Walter and Eliza Hall Institute of Medical Research, Melbourne, VIC, Australia 3052.
[6]Department of Medical Biology, The University of Melbourne, Parkville, VIC, Australia 2010.
[7]Present address: Departments of Developmental Biology and Neuroscience, Washington University School of Medicine, St. Louis, MO 63110, USA.

[*]To whom correspondence may be addressed. Email: gregm@uq.edu.au.

Supplementary Information

Supplementary figures

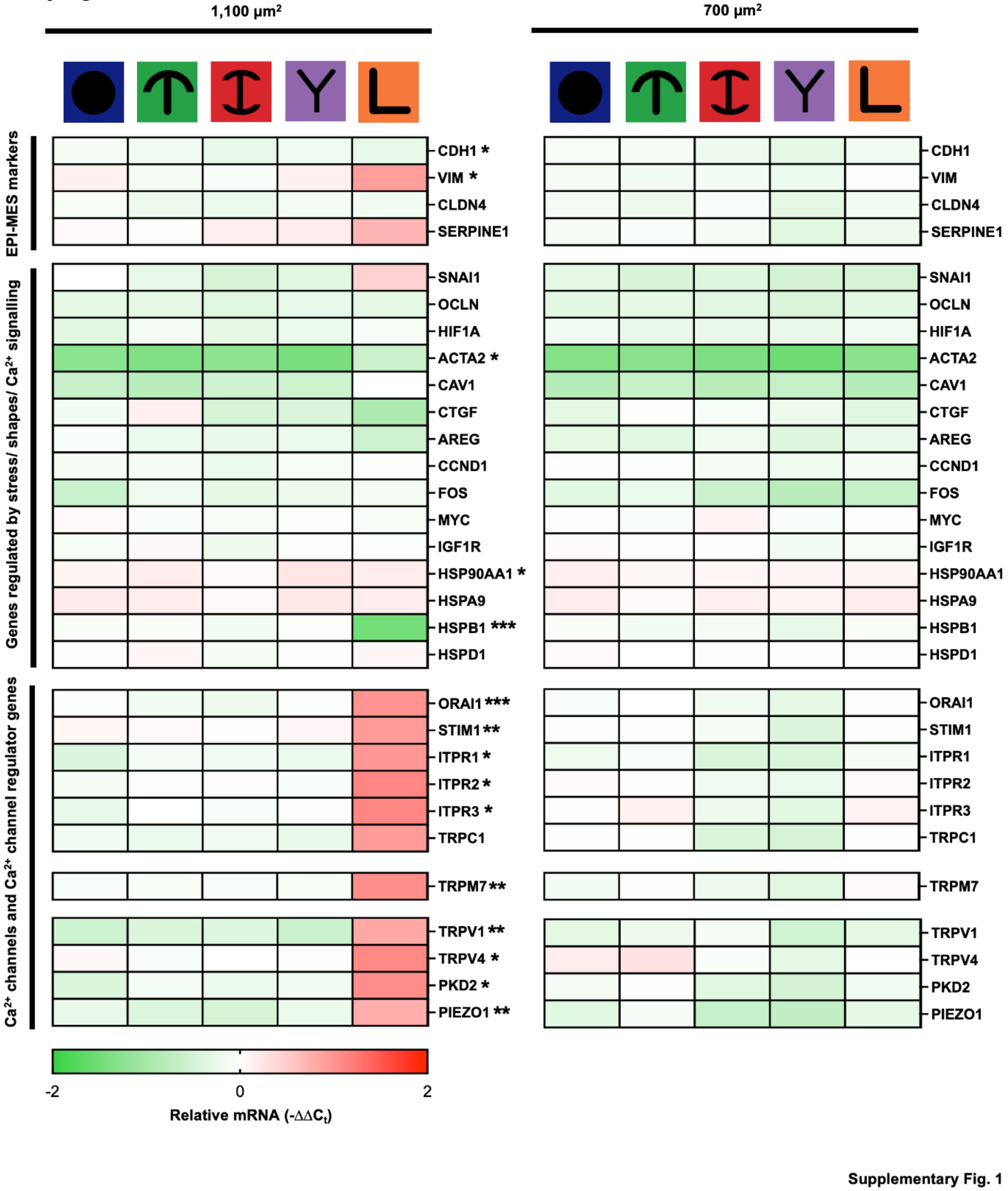

Supplementary Fig. 1: 1,100 μm² "L" micropatterns changes the expression of EPI-MES and Ca^{2+} signalling genes in MCF-7 epithelial like breast cancer cells.

Heatmap of expression changes ($-\Delta\Delta C_t$) including EPI-MES markers, Ca^{2+} channels and Ca^{2+} channel regulators and other genes on 1,100 μm² (left) or 700 μm² (right) micropatterned plates. Data represent the average gene expression changes normalized to unpatterned wells ($-\Delta\Delta C_t$) (n = 4 biological replicates, except *TRPV1* n = 3 biological replicates). Unmarked $P \geq 0.05$, * $P < 0.05$, ** $P < 0.01$, *** $P < 0.001$ ("O" vs "L").

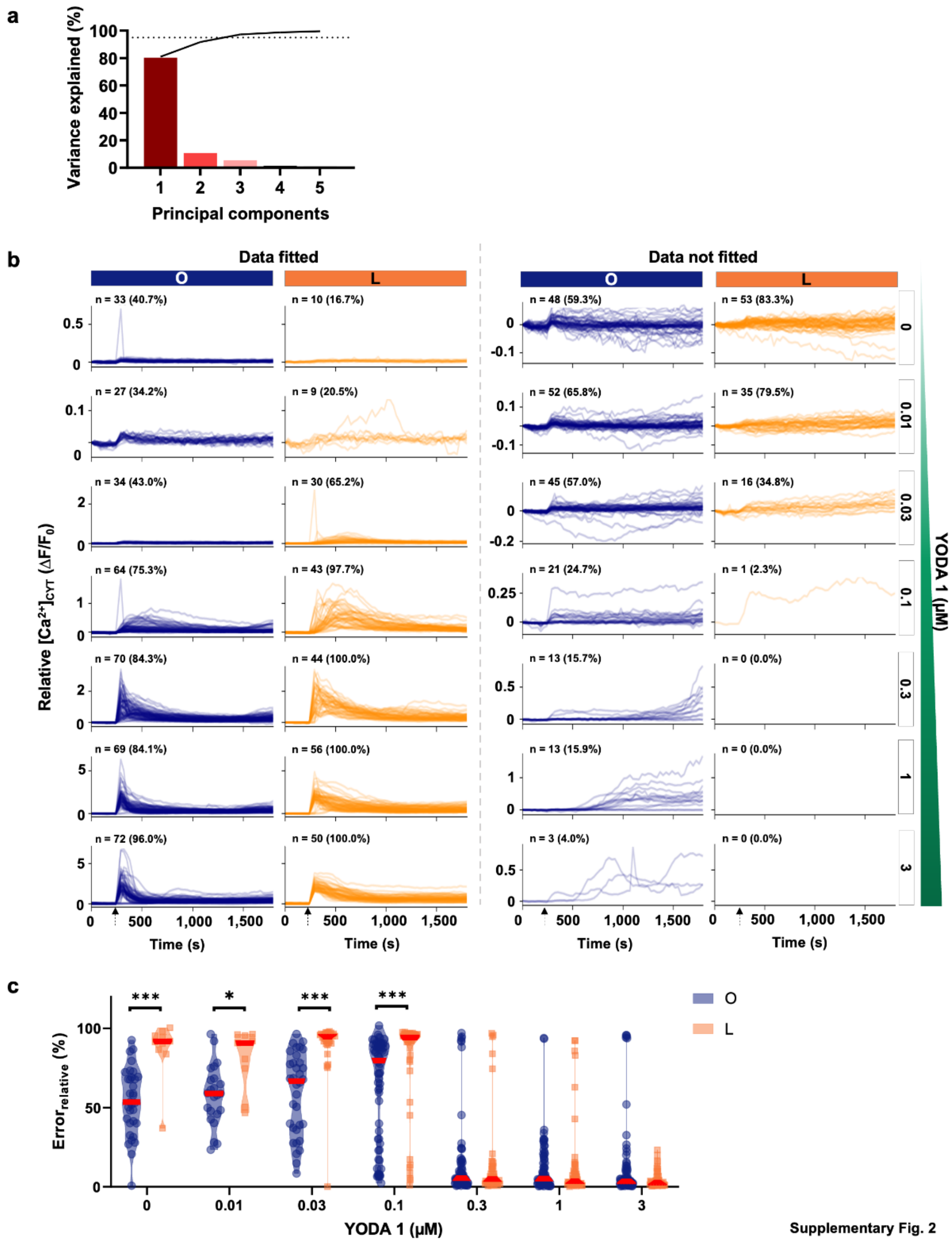

Supplementary Fig. 2: Principal components analysis and time series modelling further defines cell shape-dependent changes in YODA 1-induced Ca^{2+} influx.

(a) Principal component analysis (PCA) reveals that over 95% of the variance in the time series over both shapes and all concentrations can be explained by the top three components (PC1, PC2, PC3). Black curve shows the cumulative variance explained. Horizontal dotted line represents the percentage variance explained = 95%. **(b)** Fitting of nonlinear model for the quantification of Ca^{2+} decay time: data that meet the criteria for model fitting (left); data that do not meet the criteria for model fitting (right). The number of time series modelled are annotated as an n-value and percentage of the total sample. Refer to Supplementary Information for fitting criteria. **(c)** Model error for each cell at different YODA 1 concentrations. Scatter and violin plots for the sum of squared residuals (r^2 error) relative to the variance of the data for each shape and concentration. Unmarked P ≥ 0.05, * P < 0.05, *** P < 0.001 ("O" vs "L").

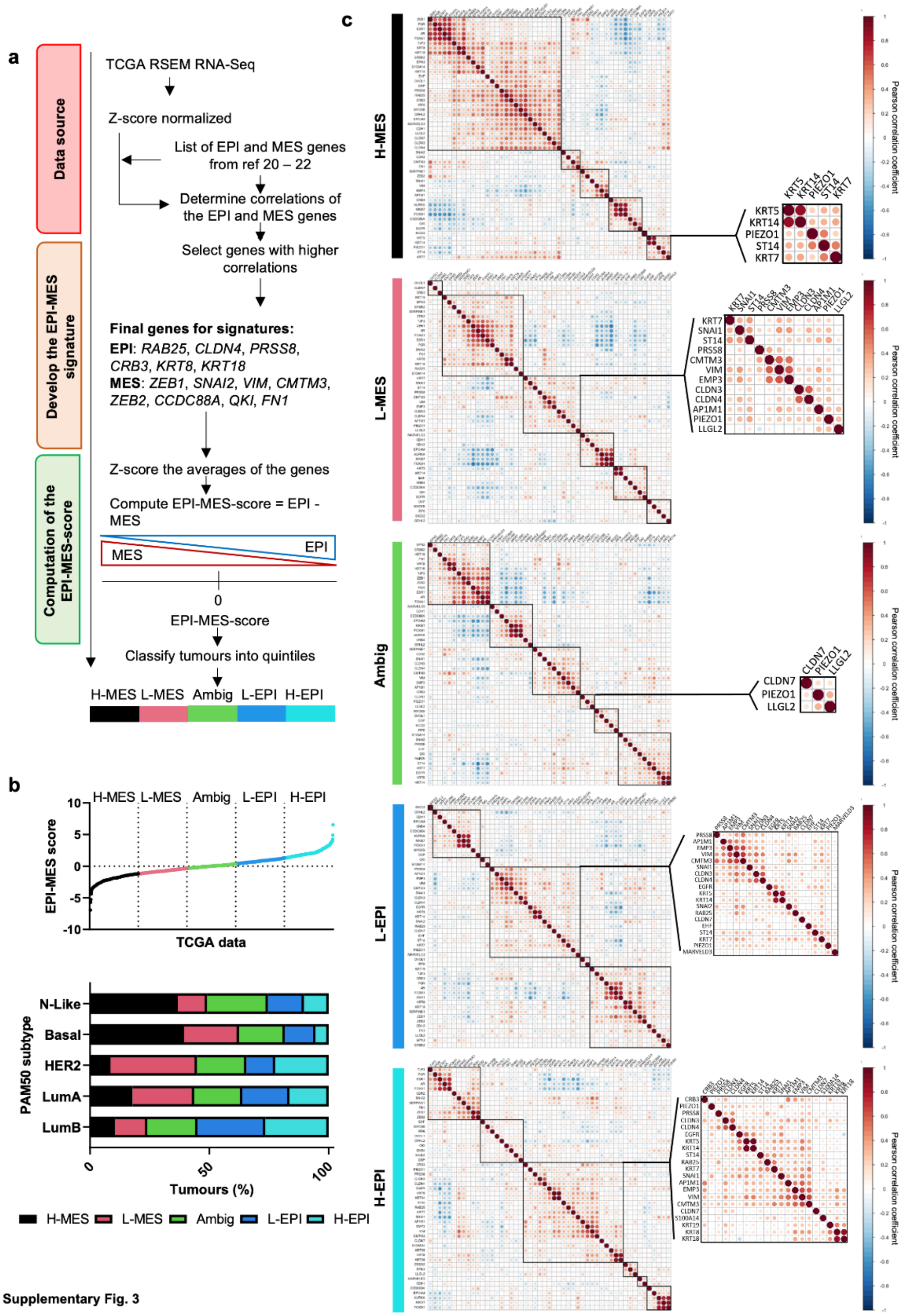

Supplementary Fig. 3: The development and validation of the EPI-MES signature.
(a) Schematic representation for the development of the EPI-MES signature. **(b)** Classification of the TCGA breast tumor dataset into quintiles based on the EPI-MES score (top) and the quintile distribution within each of the PAM50 molecular intrinsic subtypes (bottom). **(c)** Correlation profile changes with the EPI-MES phenotype. Correlation of *PIEZO1* and the EPI-MES marker genes in high-mesenchymal (H-MES), low-mesenchymal (L-MES), ambiguous (Ambig), low-epithelial (L-EPI) and high-epithelial (H-EPI) quintiles. Insets show genes grouped with *PIEZO1* based on hierarchical clustering for each of the EPI-MES quintiles.

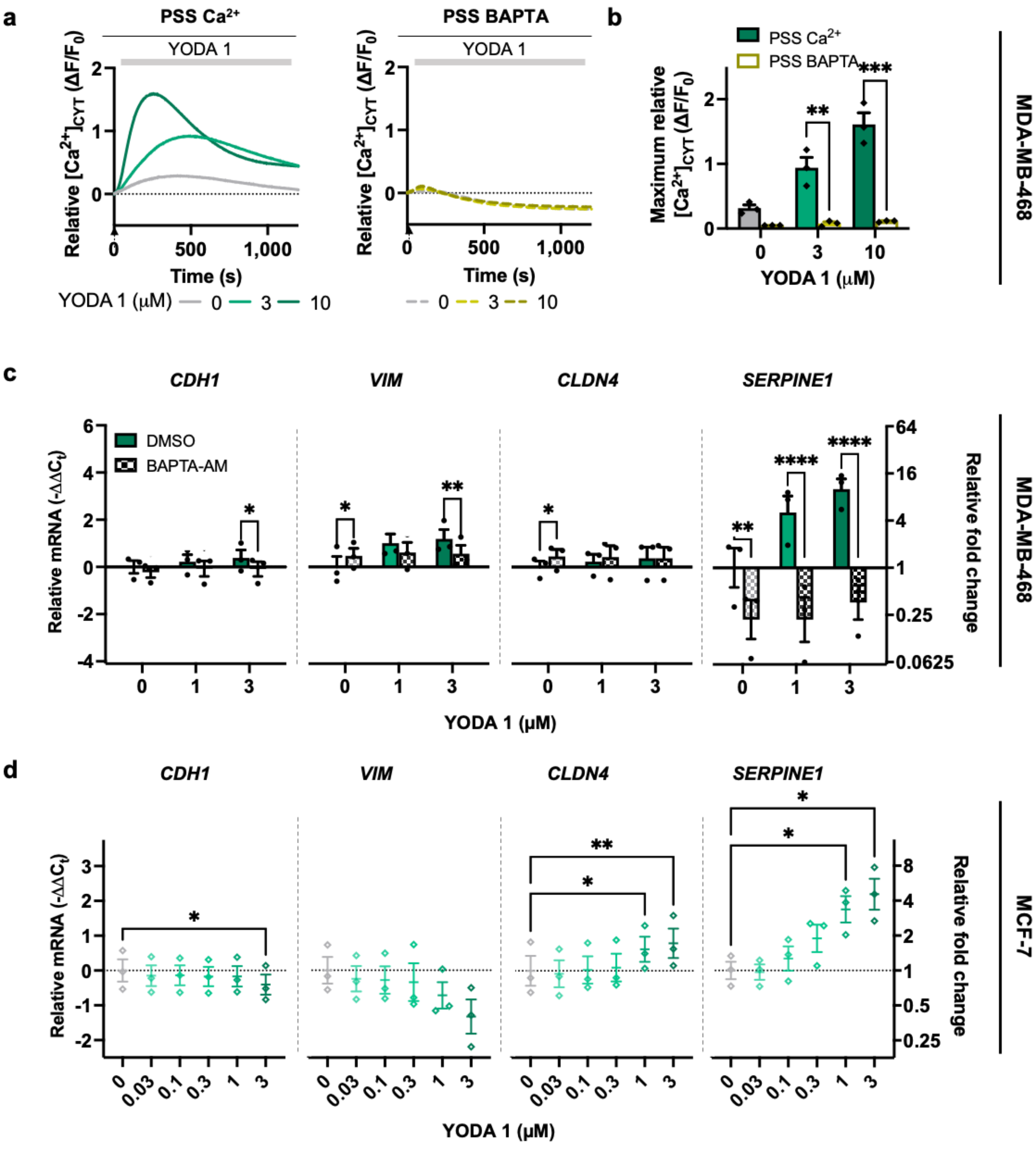

Supplementary Fig. 4

Supplementary Fig. 4: YODA 1-mediated Ca^{2+} influx induces changes in the expression of specific EPI-MES markers.

(a) YODA 1-induced $[Ca^{2+}]_{CYT}$ increases are through influx from extracellular Ca^{2+}. Media free of extracellular Ca^{2+} (PSS BAPTA) eliminates YODA 1-induced increases in $[Ca^{2+}]_{CYT}$ in MDA-MB-468 breast cancer cells. **(b)** Maximum relative $[Ca^{2+}]_{CYT}$ ($\Delta F/F_0$) induced by YODA 1 in media with (PSS Ca^{2+}) and without extracellular Ca^{2+} (PSS BAPTA). **(c)** Assessment of mRNA expression of *CDH1*, *VIM*, *CLDN4* and *SERPINE1* in MDA-MB-468 cells in the presence of YODA 1 with and without BAPTA-AM (6 h). **(d)** Alterations in mRNA expression of *CDH1*, *VIM*, *CLDN4* and *SERPINE1* in MCF-7 cells induced by YODA 1 (24 h). Where appropriate, data shown represent the mean ± S.E.M. (n = 3 biological replicates). Unmarked $P \geq 0.05$, * $P < 0.05$, ** $P < 0.01$, *** $P < 0.001$.

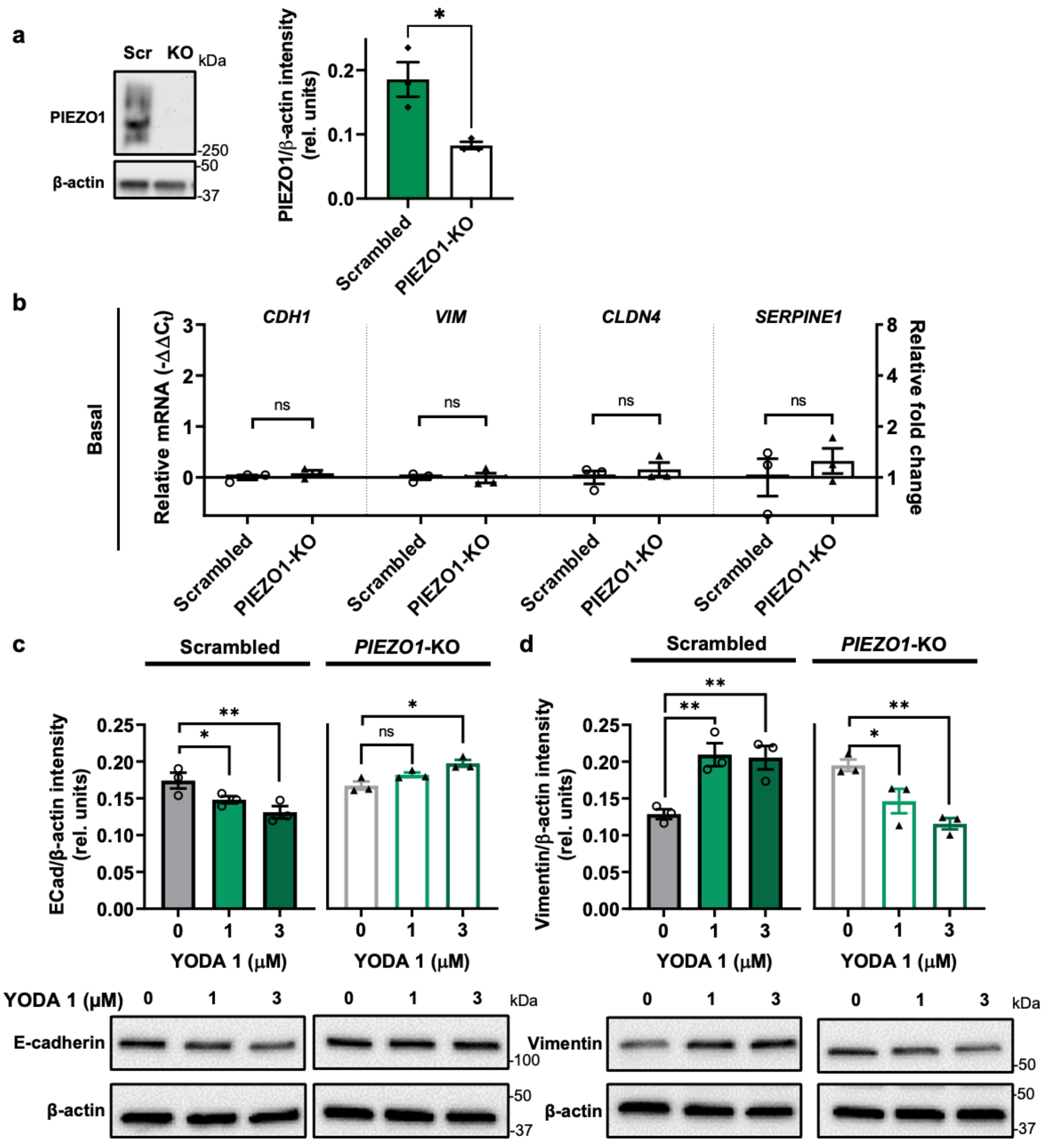

Supplementary Fig. 5

Supplementary Fig. 5: The effect of CRISPR-Cas9-mediated *PIEZO1* knockout on basal EPI-MES marker expression and YODA 1-mediated protein expression changes.
(a) Densitometry and representative immunoblot showing knockdown of *PIEZO1* in MDA-MB-468 cells. **(b)** CRISPR-Cas9-mediated *PIEZO1* knockout does not change the basal mRNA expression of *CDH1, VIM*, *CLDN4* and *SERPINE1* in MDA-MB-468 cells. **(c)** Densitometry and representative immunoblot showing knockdown of *PIEZO1* eliminates the downregulation of the epithelial marker, E-cadherin, in MDA-MB-468 by YODA 1 (72 h). **(d)** Densitometry and representative immunoblot showing knockdown of *PIEZO1* changes YODA 1-mediated induction of the mesenchymal marker vimentin in MDA-MB-468 cells (24 h). β-actin was used as the loading control. Data shown represent the mean ± S.E.M. (n = 3 biological replicates). ns P ≥ 0.05, * P < 0.05, ** P < 0.01.

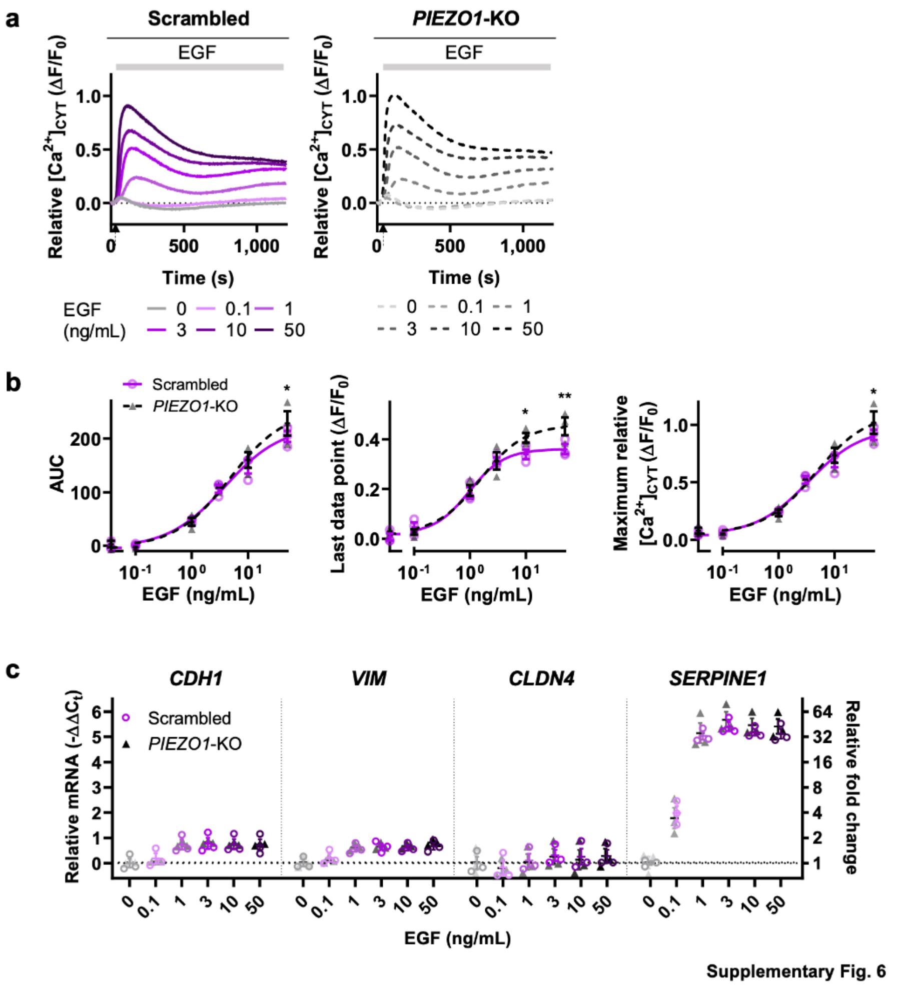

Supplementary Fig. 6: CRISPR-Cas9-mediated *PIEZO1* knockout does not abolish EGF-mediated changes in Ca^{2+} and gene expression in breast cancer cells.
(a) Knockdown of *PIEZO1* does not eliminate epidermal growth factor (EGF)-induced increases in $[Ca^{2+}]_{CYT}$ in MDA-MB-468 breast cancer cells. **(b)** $[Ca^{2+}]_{CYT}$ area under the curve (AUC) (left), sustained $[Ca^{2+}]_{CYT}$, last data point ($\Delta F/F_0$) (middle) and maximum relative $[Ca^{2+}]_{CYT}$ change ($\Delta F/F_0$) (right) for cells induced by different concentrations of EGF (0 – 50 ng/mL). **(c)** CRISPR-Cas9-mediated *PIEZO1* knockout does not abolish EGF-mediated changes in gene expression in breast cancer cells. mRNA expression of *CDH1, VIM*, *CLDN4* and *SERPINE1* in MDA-MB-468-*PIEZO1*-KO cells and MDA-MB-468-scrambled cells stimulated by EGF (6 h). Data shown represent the mean ± S.E.M. (n = 3 biological replicates). Unmarked $P \geq 0.05$, * $P < 0.05$, ** $P < 0.01$.

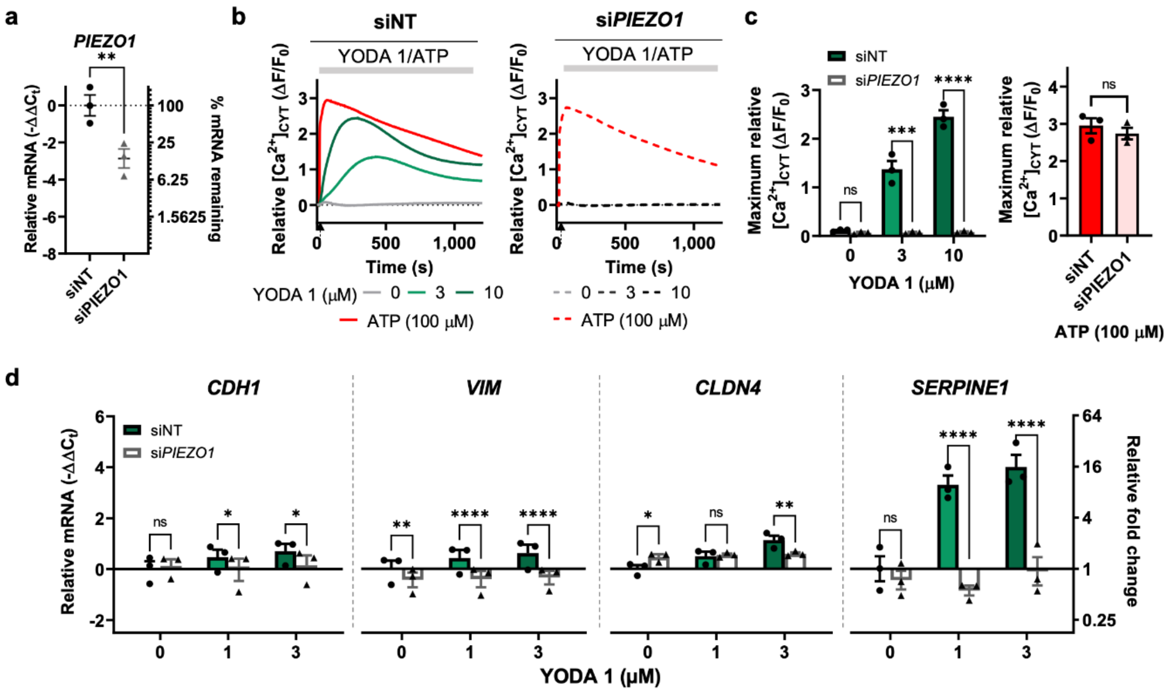

Supplementary Fig. 7

Supplementary Fig. 7: siRNA-mediated silencing of *PIEZO1* abolishes YODA 1-mediated changes in gene expression in breast cancer cells.

(a) Confirmation of *PIEZO1* siRNA-mediated silencing using qRT-PCR. (b) *PIEZO1* siRNA-mediated silencing eliminates YODA 1, but not ATP-induced increases in $[Ca^{2+}]_{CYT}$ in MDA-MB-468 breast cancer cells. (c) Maximum relative $[Ca^{2+}]_{CYT}$ ($\Delta F/F_0$) induced by YODA 1 (0, 3, 10 µM) (left) and 100 µM ATP (right). (d) mRNA expression of *CDH1, VIM*, *CLDN4* and *SERPINE1* in siNT and si*PIEZO1* MDA-MB-468 cells stimulated by YODA 1 (3 h). Where appropriate, data shown represent the mean ± S.E.M. (n = 3 biological replicates). ns $P \geq 0.05$, * $P < 0.05$, ** $P < 0.01$, *** $P < 0.001$, **** $P < 0.0001$.

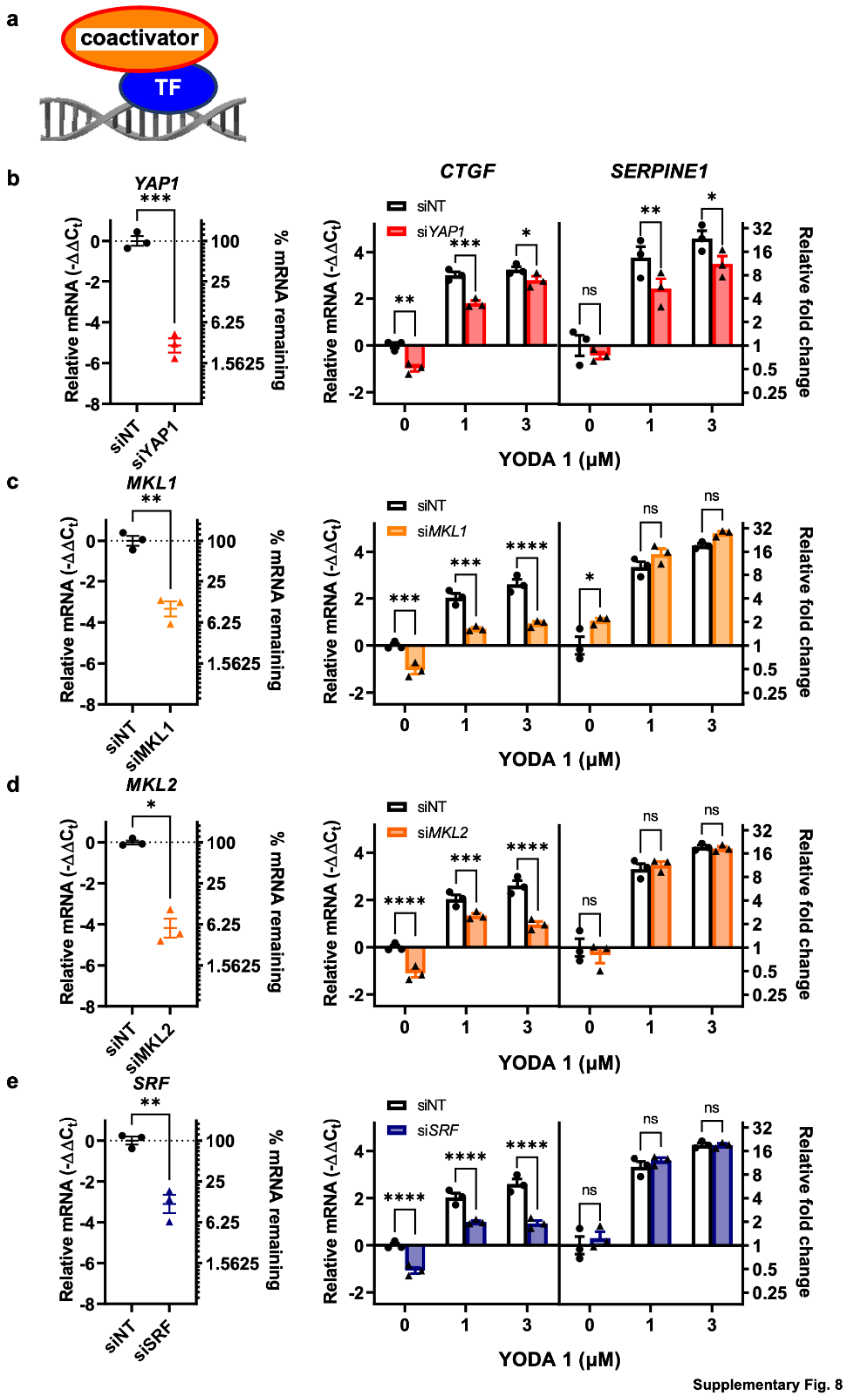

Supplementary Fig. 8: Assessment of mechanosensitive transcription factors and co-activators in YODA 1-induced changes in gene expression.
(a) Graphical illustration of the binding of mechanosensitive transcription factors and co-activators. (b) mRNA expression of *CTGF* and *SERPINE1* in MDA-MB-468 cells after siRNA-mediated silencing of *YAP1* using Dharmacon siGENOME *YAP1* siRNA. (c) mRNA expression of *CTGF* and *SERPINE1* in MDA-MB-468 cells after ON-TARGETplus SMARTpool siRNA-mediated silencing of *MKL1* (encodes for MRTF-A), (d) *MKL2* (encodes for MRTF-B) and (e) *SRF*. Data shown represent the mean ± S.E.M. (n = 3 biological replicates). ns P ≥ 0.05, * P < 0.05, ** P < 0.01, *** P < 0.001, **** P < 0.0001.

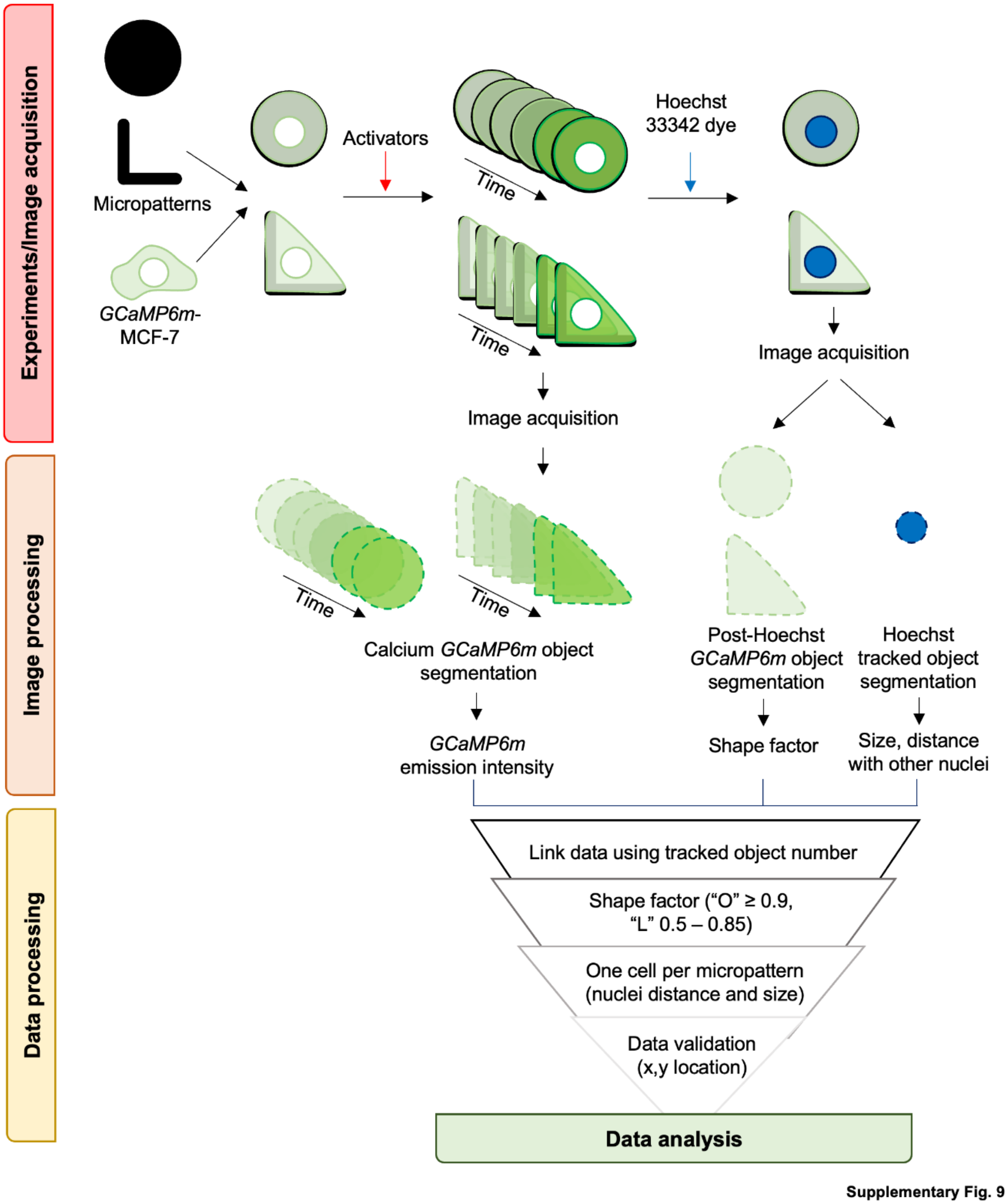

Supplementary Fig. 9: High content imaging and analysis assessing remodelling of Ca^{2+} influx associated with specific MCF-7 cell morphologies.
Schematic representation of the assessment of intracellular Ca^{2+} fluorescence in GCaMP6m-MCF-7 cells adopting specific morphologies.

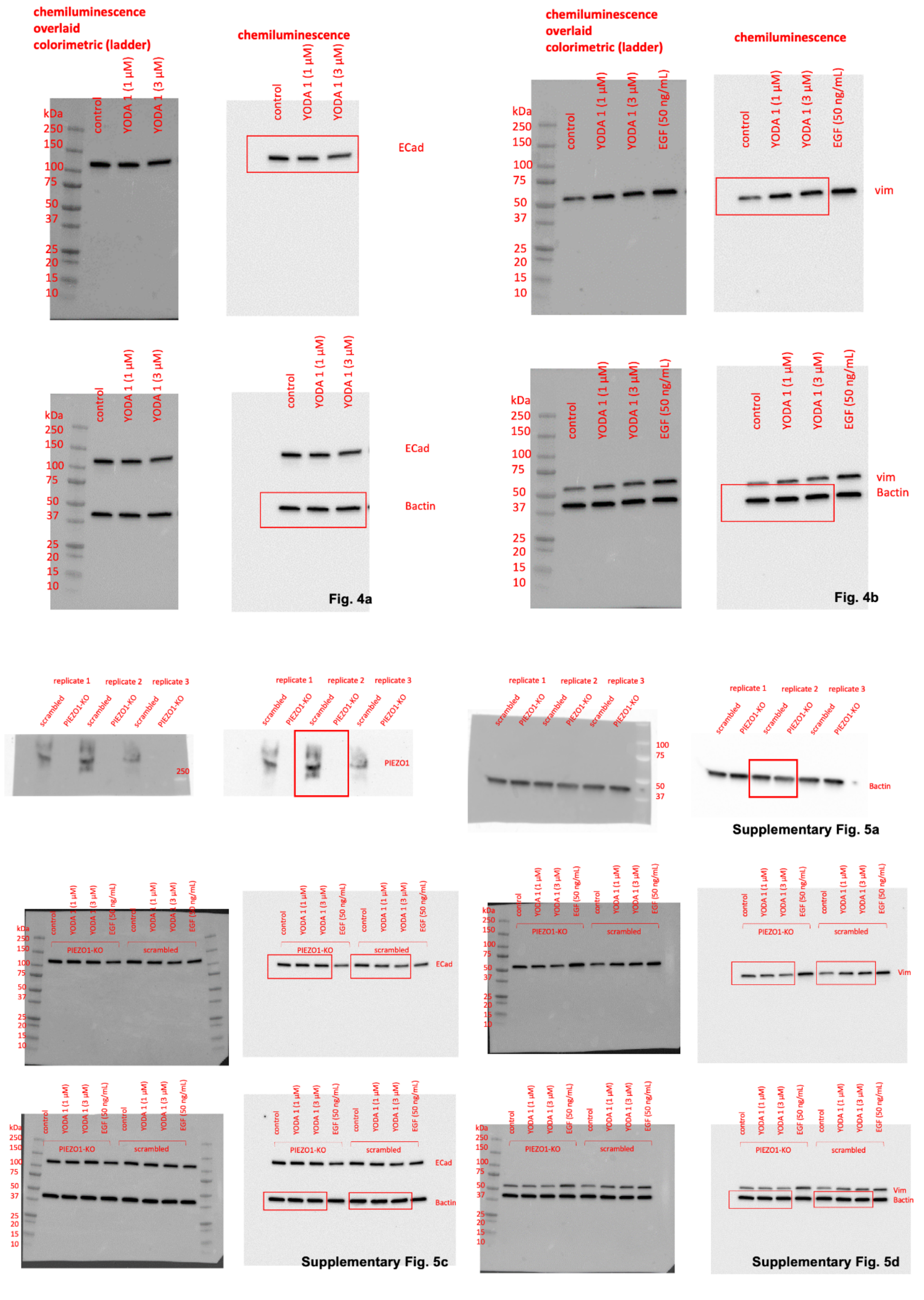

Supplementary Fig. 10

Supplementary Fig. 10: Uncropped and unedited blot images.

Supplementary table

Supplementary Table 1: Deconvolution analysis of the comparison of the genomic PCR products from MDA-MB-468-*PIEZO1*-KO cells compared to MDA-MB-468-scrambled cells using the ICE Synthego software.

**Passage**	**Indel (%)**	**Model fit to control (R^2)**	**Knockdown score**
**6**	**89**	**0.9**	**80**
**9**	**90**	**0.91**	**83**
