# Supplementary material for: Cellular geometry and epithelial-mesenchymal plasticity intersect with PIEZO1 in breast cancer cells
Source: Commun Biol. 2024 Apr 17;7:467. doi: 10.1038/s42003-024-06163-z (PMC11024093; doi:10.1038/s42003-024-06163-z)
Supplement: Supplementary file 3 — Description of Supplementary Materials [file 42003_2024_6163_MOESM3_ESM.docx]

**Description of Additional Supplementary Files**

**File name:** Supplementary Data 1

**Description:** The source data behind the graphs in Fig. 1.

**File name:** Supplementary Data 2

**Description:** The source data behind the graphs in Fig. 2.

**File name:** Supplementary Data 3

**Description:** The source data behind the graphs in Fig. 3 and Supplementary Fig. 3.

**File name:** Supplementary Data 4

**Description:** The source data behind the graphs in Fig. 4.

**File name:** Supplementary Data 5

**Description:** The source data behind the graphs in Fig. 5.
