# Supplementary material for: Cellular geometry and epithelial-mesenchymal plasticity intersect with PIEZO1 in breast cancer cells
Source: Commun Biol. 2024 Apr 17;7:467. doi: 10.1038/s42003-024-06163-z (PMC11024093; doi:10.1038/s42003-024-06163-z)
Supplement: Supplementary file 9 — Reporting Summary [file 42003_2024_6163_MOESM9_ESM.pdf]

Corresponding author(s): Gregory R. Monteith

Last updated by author(s): May 4, 2023

Reporting Summary

Nature Portfolio wishes to improve the reproducibility of the work that we publish. This form provides structure for consistency and transparency in reporting. For further information on Nature Portfolio policies, see our Editorial Policies and the Editorial Policy Checklist.

Statistics

For all statistical analyses, confirm that the following items are present in the figure legend, table legend, main text, or Methods section.

n/a	Confirmed	
☐	☒	The exact sample size (*n*) for each experimental group/condition, given as a discrete number and unit of measurement
☐	☒	A statement on whether measurements were taken from distinct samples or whether the same sample was measured repeatedly
☐	☒	The statistical test(s) used AND whether they are one- or two-sided<br>*Only common tests should be described solely by name; describe more complex techniques in the Methods section.*
☒	☐	A description of all covariates tested
☐	☒	A description of any assumptions or corrections, such as tests of normality and adjustment for multiple comparisons
☐	☒	A full description of the statistical parameters including central tendency (e.g. means) or other basic estimates (e.g. regression coefficient) AND variation (e.g. standard deviation) or associated estimates of uncertainty (e.g. confidence intervals)
☐	☒	For null hypothesis testing, the test statistic (e.g. F, t, r) with confidence intervals, effect sizes, degrees of freedom and P value noted<br>*Give P values as exact values whenever suitable.*
☒	☐	For Bayesian analysis, information on the choice of priors and Markov chain Monte Carlo settings
☒	☐	For hierarchical and complex designs, identification of the appropriate level for tests and full reporting of outcomes
☐	☒	Estimates of effect sizes (e.g. Cohen's *d*, Pearson's *r*), indicating how they were calculated

Our web collection on statistics for biologists contains articles on many of the points above.

Software and code

Policy information about availability of computer code

Data collection	MetaXpress (version 6.2.3.733) was used for ImageXpress Micro high content imaging.<br>JuLi Stage (version 1.0.4.2.2) was used for JuLi Stage brightfield imaging.<br>StepOne Software (version 2.3) was used for real-time qPCR.<br>Image Lab Touch Software (version 2.3.0.07) was used for ChemiDoc immunoblot imaging.<br>ScreenWorks (version 2.0.0.27) was used for fluorometric imaging plate reader FLIPR cytosolic calcium imaging.
Data analysis	MetaXpress (version 6.2.3.733) was used for high content image analysis. CSV files were exported and processed using R (version 3.5.1).<br>Brightfield images were exported into ImageJ (version 1.48v).<br>Densitometry for immunoblots were performed with the Image Lab Software (version 5.2.1).<br>Where appropriate, data were tabulated on Excel and visualized and analyzed using R (version 3.5.1) or GraphPad Prism (version 7.01 or 9.0 for Windows).<br>PCA and nonlinear model fitting analyses were performed with Python (version 3.9.5) in Jupyter Lab (version 3.0.16) with packages Matplotlib (version 3.4.2), NumPy (version 1.21.0), pandas (version 1.2.5), scikit-learn (version 0.24.2), SciPy (version 1.6.3), statsmodels (version 0.12.2).<br>Breast tumour correlation analysis was performed with R (version 4.1.2).

For manuscripts utilizing custom algorithms or software that are central to the research but not yet described in published literature, software must be made available to editors and reviewers. We strongly encourage code deposition in a community repository (e.g. GitHub). See the Nature Portfolio guidelines for submitting code & software for further information.

Data

Policy information about availability of data

All manuscripts must include a data availability statement. This statement should provide the following information, where applicable:

- Accession codes, unique identifiers, or web links for publicly available datasets
- A description of any restrictions on data availability
- For clinical datasets or third party data, please ensure that the statement adheres to our policy

The datasets generated and/or analyzed during the current study are available from the corresponding author on reasonable request.

Field-specific reporting

Please select the one below that is the best fit for your research. If you are not sure, read the appropriate sections before making your selection.

☒ Life sciences ☐ Behavioural & social sciences ☐ Ecological, evolutionary & environmental sciences

For a reference copy of the document with all sections, see nature.com/documents/nr-reporting-summary-flat.pdf

Life sciences study design

All studies must disclose on these points even when the disclosure is negative.

Sample size	All experiments were performed across at least three independent experiments/cell passages. Number of single micropatterned cells analyzed for principal components analysis and time series modelling were indicated in Supplementary Fig. 2.
Data exclusions	Micropatterned regions with more than one cell were excluded from high content intracellular calcium fluorescence image analysis as detailed in the Methods section. Criteria for data exclusion from fitting of nonlinear model for the quantification of calcium decay time are detailed in the Methods. No other data were excluded from analysis.
Replication	All replication attempts were successful.
Randomization	Experiments were conducted according to planned protocols and were not randomized. All experiments were performed across at least three independent experiments/cell passages to ensure reproducibility.
Blinding	Investigators were not blinded during data collection. Blinding was not necessary as data acquisition used automated systems such as automated epifluorescence microscope and StepOne Plus Real-Time PCR System or not feasible when the different concentrations of the activators must be labelled. Blinding was also not necessary as data analysis used automated image analysis pipeline or not feasible when the effect was concentration dependent.

Reporting for specific materials, systems and methods

We require information from authors about some types of materials, experimental systems and methods used in many studies. Here, indicate whether each material, system or method listed is relevant to your study. If you are not sure if a list item applies to your research, read the appropriate section before selecting a response.

Materials & experimental systems

n/a	Involved in the study
☐	☒ Antibodies
☐	☒ Eukaryotic cell lines
☒	☐ Palaeontology and archaeology
☒	☐ Animals and other organisms
☒	☐ Human research participants
☒	☐ Clinical data
☒	☐ Dual use research of concern

Methods

n/a	Involved in the study
☒	☐ ChIP-seq
☒	☐ Flow cytometry
☒	☐ MRI-based neuroimaging

Antibodies

Antibodies used	Sigma, V6389, Anti-Vimentin antibody, Mouse monoclonal, clone V9, lot 118K4801<br>Cell Signaling, #14472, E-Cadherin (4A2), Mouse monoclonal, clone 4A2, lot 7<br>Sigma, A5441, Anti-β-Actin antibody, Mouse monoclonal, clone AC-15, lot 079M4799V<br>Bio-rad, #1706516, Goat Anti-Mouse IgG (H + L)-HRP Conjugate, lot 64040709<br>Santa Cruz, sc-101199, Anti-YAP1 antibody (63.7), Mouse monoclonal, clone 63.7, lot I1520

	Cell Signaling, #4408, Anti-mouse IgG (H+L), F(ab')2 Fragment (Alexa Fluor® 488 Conjugate), lot 3<br>Novus, NBP2-75617, PIEZO1 Antibody (2-10), Mouse monoclonal, clone 2-10, lot H660661018
Validation	https://www.sigmaaldrich.com/AU/en/product/sigma/v6389<br>https://www.cellsignal.com/products/primary-antibodies/e-cadherin-4a2-mouse-mab/14472<br>https://www.sigmaaldrich.com/AU/en/product/sigma/a5441<br>https://www.bio-rad.com/en-au/sku/1706516-goat-anti-mouse-igg-h-l-hrp-conjugate?ID=1706516<br>https://www.scbt.com/p/yap-antibody-63-7<br>https://www.cellsignal.com/products/secondary-antibodies/anti-mouse-igg-h-l-f-ab-2-fragment-alexa-fluor-488-conjugate/4408<br>https://www.novusbio.com/products/piezo1-antibody-2-10_nbp2-75617

Eukaryotic cell lines

Policy information about cell lines

Cell line source(s)	MCF-7 (ATCC HTB-22) and HEK293T (ATCC CRL-3216) cell lines were sourced from ATCC. The MDA-MB-468 cell line was from The Brisbane Breast Bank, UQCCR, Australia.
Authentication	MCF-7 (last authenticated September 2018), GCaMP6m-MCF-7 (last authenticated Feb 2019) and MDA-MB-468 (last authenticated July 2021) cell lines were routinely authenticated by short tandem repeat (STR) profiling.
Mycoplasma contamination	All cell lines were routinely tested for mycoplasma contamination; results for mycoplasma contamination were negative.
Commonly misidentified lines<br>(See ICLAC register)	No cell line used in this study was listed as commonly misidentified lines (ICLAC version 11).
